# Biosynthesis and Anti-Mycotoxigenic Activity of *Zingiber officinale* Roscoe-Derived Metal Nanoparticles

**DOI:** 10.3390/molecules26082290

**Published:** 2021-04-15

**Authors:** Mohamed Raafat, Ashraf S. A. El-Sayed, Manal T. El-Sayed

**Affiliations:** 1 Department of Pharmacology and Toxicology, College of Pharmacy, Umm Al-Qura University, Makkah 21955, Saudi Arabia; maabdalla@uqu.edu.sa; 2 Botany and Microbiology Department, Faculty of Science, Zagazig University, Zagazig 44519, Egypt

**Keywords:** biological activity, characterization, metals nanoparticles, ochratoxin A, *Zingiber officinale*

## Abstract

Mycotoxigenic fungi have attracted special attention due to their threat to food security and toxicity to human health. Aqueous extract of *Zingiber officinale* Roscoe was used as reducing and capping agent for the synthesis of silver (AgNPs), copper (CuNPs), and zinc oxide (ZnONPs) nanoparticles. UV-Visible spectra of the AgNPs, CuNPs, and ZnONPs showed absorption peaks at λ_max_ 416 nm, 472 nm, and 372 nm, respectively. Zeta potential of AgNPs, CuNPs, and ZnONPs were −30.9, −30.4 and −18.4 mV, respectively. ZnONPs showed the highest activity against *Aspergillus awamori* ZUJQ 965830.1 (ZOI 20.9 mm and MIC 24.7 µg/mL). TEM micrographs of ZnONPs-treated *A. awamori* showed cracks and pits in the cell wall, liquefaction of the cytoplasmic content, making it less electron-dense. The sporulation and ochratoxin A production of *A. awamori* was inhibited by ZnONPs in a concentration-dependent pattern. The inhibition percentage of OTA were 45.6, 84.78 and 95.65% for 10, 15, 20 of ZnONPs/mL, respectively.

## 1. Introduction

Mycotoxin contamination of feed and foods is a serious threat to animal and human health due to their potential for induction of cancer, mutagenicity and estrogenic gastrointestinal, vascular, urogenital, nervous and kidney disorders. Mycotoxigenic fungi may contaminate agricultural products in the field, during storage (post-harvest spoilage), or during processing [1]. There are three common strategies for controlling mycotoxin production: fungal growth inhibition, mycotoxin absorption and risk elimination. The usage of biogenic nanoparticles (NPs) could be one of the most promising methods for preventing the occurrence of mycotoxins [2]. Nanoparticles are the bridge between bulk size materials and atomic structures with higher reactivity that is mainly due to a high surface-area-to-volume ratio. Due to their antimicrobial characteristics, NPs have enormous applications in nutrition, agriculture, medicine, health, and other aspects [3,4]. The antimicrobial mode of action of NPs may be due to their intrinsic ability to bind with biomolecules of interest [5], their ability to penetrate microbial cell walls [6], generation of reactive oxygen species (ROS) in microbial cells [7], modulation of microbial signal transduction pathways [8] and their oligodynamic effect [9].

Nanoparticle synthesis commonly relies on chemical reduction that can be mediated by microwave-assisted techniques, electrochemical methods, decomposition, and wet chemical procedures [10]. These methods are mainly based on the highly reactive reducing agents, for instance, formaldehyde, sodium borohydride (NaBH_4_), and hydrazine (N_2_H_4_), resulting in highly poisonous and flammable byproducts that could hinder any subsequent environmental and biological applications [11,12]. Hence, there is a clear need for alternative, cost-effective, secure, and eco-friendly NP synthesis methods. Recently, the emergence of efficient green methods has attracted much attention. Environmentally friendly nontoxic materials such as plant extracts, fungi, and bacteria offer many benefits for NP synthesis for various eco-friendly and highly biocompatible biomedical applications [13]. Microbial enzymes or phytochemicals with antioxidant or reducing properties are usually implemented in the production of the desired NPs. The major constituents factors involved in the preparation of NPs using biological methods are the solvent medium for the synthesis, a nontoxic stabilizing agent, and an environmentally friendly reducing agent [14].

The phytosynthesis of NPs by plants is a single-step process (not like microbial isolation, culturing, and preservation) and reproducible that often results in more stable NPs. Secondary metabolites in plant systems such as terpenoids (eugenol), flavonoids (luteolin, quercetin), sugars, alkaloids, amino acids (tryptophan and tyrosine), phenolic compounds, proteins, energy or electrons released during glycolysis, are the major paths for the phytosynthesis of NPs, and could be act as NP stabilizers, preventing their aggregation [15,16]. Extracts of different plant tissues have been utilized for the synthesis of many NPs, as extensively reported [12,17,18,19,20,21,22,23].

Fungal poisoning of foods and feeds causes major economic losses. The most frequent mycotoxigenic genera are *Aspergillus*, *Alternaria*, *Claviceps*, *Fusarium*, *Penicillium*, and *Stachybotrys* [1]. Ochratoxin A (OTA) is a nephrotoxic, carcinogenic, teratogenic, immunotoxic and hepatotoxic toxin. *A. awamori* has recently described as an OTA and fumonisin-producing species [24]. *A. fumigatus* produces mycotoxins like fumagillin and viriditoxin which cause cytotoxic and apoptotic effects on human T lymphocytes and may exert immunosuppressive effects [25]. *F. oxysporum* produces toxins like zearalenone, moniliformin, and diacetoxyscirpenol and causes wilt disease. Many approaches have been investigated to either stop OTA contamination, detoxify foods contaminated with OTA or repress OTA absorption in the gastrointestinal tract [26]. Nanoparticles have recognizable activities against various mycotoxigenic fungi such as *Bipolaris sorokiniana*, *Magnaporthe grisea*, *Botrytis cinerea*, *Candida albicans*, *C. tropicalis*, *Aspergillus* spp., *Fusarium* spp., *P. expansum*, and *Al. alternata* [27,28,29,30].

Medicinal plants with recognized therapeutic features and no side effects have attracted much attention. *Zingiber officinale* Roscoe (ginger) is among these plants. It belongs to the family Zingiberaceae and has been traditionally consumed as a spice with strong therapeutic activity [31]. Ginger has been widely used in ethnomedicine for the cure of several ailments. In modern phytotherapy, ginger preparations are predominantly used to counteract nausea and vomiting in pregnant women but they have anti-inflammatory, analgesic, and metabolic actions in clinical applications in knee osteoarthritis, dysmenorrhea, type-2 diabetes, hyperlipidemia, overweight, and obesity [31]. There are more than 400 different compounds in ginger which include carbohydrates, lipids, terpenes, zingiberene, β-bisabolene, α-farnesene, β-sesquiphellandrene, α-curcumin, gingerols, paradols, and shogaols [32]. In view of that, ginger is plentiful in antioxidants, and the biomolecules in the ginger extract are assumed to play a critical role in the reduction of metal ions to metallic NPs [33]. Therefore, in this study *Z. officinale*, Roscoe has been used as producers of AgNPs, CuNPs and ZnONPs and their antifungal activities against some mycotoxigenic fungi were determined.

## 2. Materials and Methods

### 2.1. Materials

Fresh rhizomes of *Z. officinal* were obtained from local markets in Zagazig, Egypt. Silver nitrate, potato dextrose agar (PDA) and yeast extract (Sigma-Aldrich, Lyon, France), copper sulfate (Alpha Chemika, Mumbai, India), zinc sulfate (Andenex-Chemie, Engelhard, UK), OTA standards, methanol, acetonitrile, chloroform (Sigma Chemical Co., St. Louis, MO, USA) and anhydrous sodium sulfate (El Nasr Pharmaceutical Chemicals Co., Cairo, Egypt) were used.

### 2.2. Preparation of Ginger Extract

Washed and peeled rhizome (5 g) of *Z. officinale* was cut into small slices and pulverized in mortar with slowly addition of 20 mL deionized water then boiled for 2 min. The extract was separated using a muslin cloth and centrifuged at 6000 rpm for 20 min to eliminate large ginger fibers [34], and the extract was stored at 4 °C for further use.

### 2.3. Synthesis of AgNPs, CuNPs, and ZnONPs

To synthesize AgNPs and CuNPs, ginger extract (5 mL) was added to a 250 mL Erlenmeyer flask containing 95 mL of 1mM AgNO_3_ or 1 mM CuSO_4_·2H_2_O (freshly prepared). The mixtures were kept on an orbital shaker incubator (HYSC, Model Si-100R, Seoul, Korea) at 120 rpm, 30 °C for 24 h in the dark [35]. To synthesize ZnONPs 0.5 mL of the ginger extract was added to 50 mL of 100 mM ZnSO_4_ with stirring on a magnetic stirrer, and the pH was adjusted to 12.0 by 1.0 M NaOH, this mixture was kept on the stirrer for 2 h until a white coalescence formed [36]. Ginger extract was used as positive control and metal ions as negative control. The reduction of metal ions was routinely detected by visual inspection of the solution color. The developed NPs was obtained by centrifugation (5000 rpm for 5 min) then redispersed in deionized water. The precipitated NPs were air dried to a constant weight and re-dispersed in deionized water by ultrasonication as a stock solution. The stock solution was diluted with sterilized deionized water to prepare the required final concentrations used for further study.

### 2.4. Physical Characterization of Nanoparticles

The biosynthesized NPs were evaluated by UV–vis spectra in the range of 200–800 nm (UV-Vis spectrophotometer, T80, PG Instrument, Leicestershire, UK) after 24 h (AgNPs and CuNPs) and 72 h (ZnONPs) of reaction. The positive control (ginger extract) and negative control (metal precursor) were used as blanks.

The biosynthesized NPs were characterized by TEM (JEOL-1010, JEOL, Tokyo, Japan) connected to EDX (Model Oxford 6587 INCA x-sight, Cambridge, MA, USA) to determine the morphological and elemental composition of the particles.

To evaluate the particle electrostatic charge, 100 µL of the solution was diluted in 1.5 mL of water. Then it was poured into a Zetasizer-nano series cuvette (Nano ZS) i (Malvern, Worcestershire, UK) (Nanotechnology Centre & Advanced Materials Central Lab, NAMCL), Agriculture Research Centre, Giza, Egypt). The results are stated as zeta potential (ζ-potential).

To confirm the AgNPs’, CuNPs’ and ZnONPs’ crystalline structures, X-ray powder diffraction (XRD) analysis was performed using a drop-coated glass substrate and the data were recorded on a D8 advanced target Cukα powder diffractometer (λ= 1.5418 Å, Bruker, Madison, USA) over the range 0–80 of 2θ (Central Metallurgical & Development Institute, Helwan, Egypt).

To detect the functional groups involved in phytosynthesis of NPs, Fourier transform infrared (FTIR) spectra of the native ginger extract and AgNPs, CuNPs, and ZnONPs solutions were performed using a FTIR 1650 spectrophotometer (Perkin–Elmer, Waltham, MA, USA), Center of Microanalysis, Cairo University, Cairo, Egypt). The samples were grinded in KBr (3% *W/W*) and discs were prepared using the hydraulic press. The discs examined in the range of 400–4000 cm^−1^.

### 2.5. Antifungal Activity of Biosynthesized AgNPs, CuNPs, and ZnONPs

The mycotoxigenic fungal isolates, *Fusarium oxysporum* FR11, *Aspergillus fumigatus* JX006238, and *A. awamori* JQ695830.1 were isolated, morphologically and molecularly identified [37,38,39]. The antifungal activity of the synthesized NPs was estimated by the disc-diffusion method [40,41]. The strains were cultured on PDA slants at 28 ± 2 °C for ten days, the spores were harvested using 10 mL of sterile distilled water of 0.05% Tween 20, and the culture surface was scraped to free the fungal spores. The spore suspensions were adjusted to give a final concentration of 10^6^ conidia/mL.

To evaluate the zone of inhibition (ZOI), one mL of fungal spore suspension was seeded into 15 mL of PDA media, shaken vigorously and then poured. After medium solidification, sterilized Whatman #1 filter paper discs (6 mm diameter) were saturated with 20 μL of the different concentrations of AgNPs, CuNPs and ZnONPs, then placed on the surface of inoculated plates and incubated at 28 ± 2 °C for 72 h. The ginger extracts in addition to authentic antifungal were used as positive control. The inhibition zone was measured in mm, all the experiments were performed in triplicates and the results were represented as mean ± SD.

Minimum inhibitory concentration (MIC), the lowest concentration of NPs repressing the visible microbial growth, was estimated. To estimate MICs, different concentrations of the corresponding NPs were supplied into 250 mL Erlenmeyer flasks containing 50 mL of yeast-extract sucrose broth (YES) (2% yeast extract, 15% sucrose, in distilled water) supplied with. After autoclaving (20 min at 120 °C) and cooling, the flasks were inoculated with one mL of fungal spore suspension and incubated at 28 ± 2 °C for five days. Positive controls were considered.

### 2.6. TEM Investigation

At the end of incubation period, the control and ZnONPs-treated biomass (with sub MIC concentration) of *A. awamori* JQ695830.1 were harvested by centrifugation and washed with distilled water. The samples of fungal biomass were prepared for TEM analysis by immersing in primary fixative (2.5% glutaraldehyde buffered to pH 7.4 with 0.2 M phosphate buffer) for 3 h. Then, the samples were post-fixed in 1% osmium tetra-oxide for 2 h, buffered with phosphate buffer for 30 min. All steps of fixation were carried out at 4 °C, then, the samples were dehydrated in a series of ethanol (50% to 100%), then embedded in resin capsule [40]. The samples were sectioned into ultrathin section of 70 nm, loaded on copper grids and contrasted with uranium acetate and lead citrate before examination on a JEOL-1200 EX microscope (National Research Centre, Giza, Egypt).

### 2.7. Effect of ZnONPs on the Amount of OTA Produced by A. awamori

Different concentrations of ZnONPs (0, 10, 15 and 20 μg/mL) synthesized by *Z.* *officinale* were placed in a set of 250 mL Erlenmeyer flasks containing 50 mL of YES broth medium. The flasks were autoclaved, inoculated with 10^7^ spore suspension of *A. awamori* JQ695830.1, then incubated for 14 days at 28 ± 2 °C. For the extraction of OTA, the culture filtrate was extracted with chloroform (1:2 *v*/*v*). The chloroform phase was dried with anhydrous sodium sulfate, filtered using Whatman No.1 filter paper and evaporated on a rotary evaporator till the formation of a dry film. The precipitate was dissolved in 1 mL water: acetonitrile (3:1 *v*/*v*) and mixed well by vortexing for 30 s. The concentrations of OTA were quantitatively measured using a HPLC system (1200 Agilent series quaternary gradient pump, series 1200 autosampler, series1200 Fluorescence Detector, and HPLC 2D chemstation software (Hewlett-Packard, Les Ulis, Germany) (Animal Health Research Institute, Dokki, Egypt). The chromatographic separation performed with a reversed-phase column (Extend-C18, Zorbax column, 250 × 4.6 mm i.d., 5 μm, Agilent). The mobile phase 60:20:20 water/methanol/acetonitrile mixture used. The column temperature was adjusted to 30 °C at a flow rate 1.0 mL/min to achieve the optimum resolution of OTA. The injection volume maintained at 20 μL for both sample and standard. OTA concentrations evaluated from the standard curve using peak area for quantification.

## 3. Results and Discussion

### 3.1. Characterization of AgNPs, CuNPs, and ZnONPs

#### 3.1.1. UV-Visible Spectral Analysis

**Figure 1:**
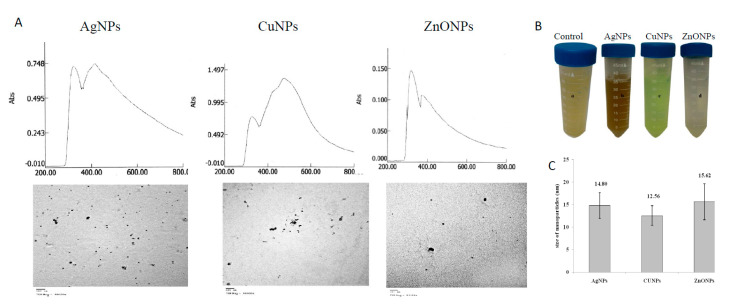
Phytosynthesis of silver nanoparticles (AgNPs), copper nanoparticles (CuNPs), and zinc oxide nanoparticles (ZnONPs) by aqueous extract of *Zingiber officinale*. (**A**), UV-Vis analyses (upper panel) and TEM images (lower panel) of the synthesized NPs. (**B**) The visual appearance of NPs. (**C**), Average sizes of synthesized NPs.

The synthesized NPs were assessed based on their UV-spectral analyses. The dark brown color, green-blue color, and white coalescent clusters indicated the bioreduction of the precursor salts AgNO_3_, CuSO4, and ZnSO_4_ to their corresponding NPs such as AgNPs, CuNPs, and ZnONPs, respectively, by the plant extract (Figure 1). The color change is a crucial indicator for the synthesis of NPs [12,31]. The developed color is due to the collective vibration of free conduction electrons induced by an interactive electromagnetic field “surface plasmon excitation (SPR) [41]. The lack of agglomeration confirms the stability and homogeneity of the synthesized NPs, suggesting the presence of stabilizing agents like polysaccharides or proteins [42]. The positive control (ginger extract) and the negative control (salt precursors) did not show any color change. UV-Vis spectra of AgNPs, CuNPs, and ZnONPs showed maximum absorption peaks at λ_max_ 416 nm, 472 nm, and 372 nm, respectively (Figure 1). Similar results were observed for synthesized AgNPs from *Carica papaya* [43], CuNPs from *Allium sativum* and *Z. officinale* [44], and ZnONPs from *Passiflora caerulea* [45]. The SPR bands that observed around ~λ326 nm, corresponding to aromatic amino residues. The metal reducing potency of ginger extract might be due to the presence of ascorbic acid and/or oxalic acid [46]. The chemical reduction converts Ag^+^ ions to Ag^0^, and evolving the nucleation of NPs, and then the NPs are confined by the layers of ascorbic acid and/or oxalic acid via electrostatic forces of the ginger extract.

#### 3.1.2. Transmission Electron Microscopy (TEM)-Analysis

From the corresponding TEM photographs, the particle sizes of AgNPs, CuNPs, and ZnONPs ranged from 10.40–19.12 nm, 7.65–15.29 nm, and 10.26–22.29 nm, with an average size of 14.80 ± 2.85 nm, 12.56 ± 2.19 nm and 15.62 ± 4.00 nm (Figure 1). The spherical NPs had edges lighter than the centers, suggesting that they were capped with biomolecules such as proteins [47]. The difference in particle size may be due to the formation of NPs at different times [48]. The morphology of synthesized NPs is directly connected to its optical and electrical characteristics [49]. Concerning to the size, the smaller-sized NPs are more effective for photothermal therapy, drug delivery, and for biological activity [35].

#### 3.1.3. Energy Dispersive X-ray Microanalysis (EDX)

**Figure 2:**
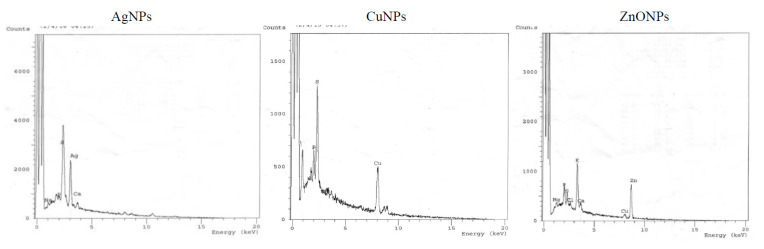
Energy dispersive X-ray microanalysis (EDX) of AgNPs, CuNPs, and ZnONPs synthesized using aqueous extract of *Zingiber officinale*.

The purity, dispersity and elemental composition of synthesized NPs were characterized by EDX. The optical absorption characteristic peaks of EDX spectra for Ag, Cu, and Zn were recorded at 3 keV, 8 keV, and 8.8 keV, respectively, (Figure 2). Similar results for EDX spectra were reported for Ag(I), Cu(II), and Zn(II), through green synthesis of NPs [50,51]. Additional peaks for Mg, P, S, K, and Ca were observed, that might be ascribed to other biomolecules in the plant extract.

#### 3.1.4. Zeta Potential (ζ-Potential) Measurements

**Figure 3:**
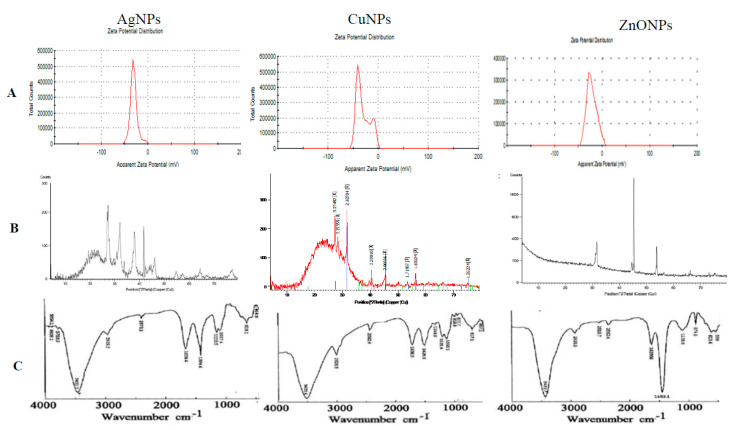
Zeta potential (ζ-Potential) (**A**), X-ray powder diffraction (XRD) (**B**), and Fourier infrared (FTIR) analyses (**C**) of AgNPs, CuNPs, and ZnONPs synthesized by aqueous extract of *Zingiber officinale*.

Zeta potential was applied for measuring the electrical charges of particles suspended in liquid, indicating the degree of repulsion/attraction between NPs. The higher ζ-potential would be the higher NPs stability [41]. Zeta potential values of AgNPs, CuNPs, and ZnONPs were −30.9, −30.4 and −18.4 mV, respectively (Figure 3). Negative ζ-potential values may be due to the negative charged functional groups of ginger biomolecules that acting as capping agents and coating the surface of the NPs. If the whole particles have a positive or negative ζ-potential, they would repel with each other, and there will be a slight tendency of the particles to agglomerate [52,53].

#### 3.1.5. XRD Analysis

From the XRD pattern of AgNPs (Figure 3) nine peaks at 26.98°, 32.19°, 37.99°, 41.79°, 46.2°, 54.66°, 57.20°, 64.34° and 77.20° of 2θ were identified as 125, 264, 111, 200, 220 and 311 reflections, respectively. The average crystal size of AgNPs was 10.9 nm. The XRD analysis is a powerful nondestructive analytical technique for identification and quantitative measurement of various crystalline characters of the particles. The sharpness of the XRD peaks means the high crystalline nature [54]. The unassigned peaks in the spectra may be due to the presence of organic matters in the ginger extract. XRD pattern of CuNPs (Figure 3) showed the existence of peaks at 27.21°, 31.52°, 40.44°, 45.37°, 56.28°, and 77.18° of 2θ which belong to 110, 111,200, and 220 planes, respectively. The average particle size of CuNPs was 10.4 nm, that consistent with those reported copper for oxide nanoparticles from peanut plant leaves [55]. XRD pattern of ZnONPs (Figure 3) showed five peaks; one very intense at 45.38° and four less intense peaks at 31.14°, 31.73°, 44.55°, and 53.75° of 2θ which belong to (100), (102), and (110) reflections, respectively. Similar XRD patterns for CuNPs and ZnONPs were reported [31,56].

#### 3.1.6. Fourier Transform Infrared Spectroscopy (FTIR)

The FTIR spectra for both the ginger extract as well for the synthesized NPs were recorded (Figure 3). The peaks in synthesized NPs and ginger extract corresponding to aromatic compounds such as terpenoids and flavonoids might be involved in the synthesis of NPs [42]. New peaks at 3854.04 and 3750.87 cm^−1^ (AgNPs) assigned to stretching of the O–H bond (possibly arising from the carbohydrates, proteins or some adsorbed water in the sample) and amide N–H stretching, respectively. A band at 3423.03 cm^−1^ with an increase in the intensity is characteristic of O–H stretching and N–H stretching mode of amides I. The slight shift at 2930.31 cm^−1^ assigned to CH_2_ asymmetric stretching; mainly lipid and protein [55]. The new peaks at 919.88 cm^−1^ (CuNPs) and 2515.69 cm^−1^ (ZnONPs) were due to –SH stretching and bending mode. The strong shift to a higher wave number at 2334.41 cm^−1^ (∆ 35 cm^−1^, AgNPs, and ∆ 27 cm^−1^, ZnONPs and CuNPs) indicated the importance of nitrogen compounds (showing triple or cumulative double bonds such as nitriles and cyanates) and sulfur compounds. A shift at 1633.41 cm^−1^ attributed to bending vibrations of amide I and amide II bands of the proteins and phenolic groups of tyrosine and tryptophan in ginger extract, revealed the presence of alkenes and aromatic compounds [57,58]. The disappearance of peak at 1575.56 cm^−1^ (in all NPs) assigned to amide II and NH deformation mode was observed. The very marked shift at 1405.85 cm^−1^ to 1384.64 cm^−1^ (AgNPs, ∆ 21 cm^−1^), 1429.95 cm^−1^ (CuNPs, ∆ 26 cm^−1^), and 1453.1 cm^−1^ (ZnONPs, ∆ 48 cm^−1^) reveals the role of –C(CH_3_)_2_ stretching in proteins and binding of metal ions to carboxylic acid groups of amino acids. The new bands at 1244.83 cm^−1^ (CuNPs) were assigned to C–O stretching (ethers)/C–N stretching (amines). The disappearance of the peak at 1312.32 cm^−1^ (in all NPs) and the new band at 874.56 (ZnONPs) and 870.70 cm^−1^ (CuNPs) are strong signals of phosphorous, P = S stretching, and heterocyclic compounds of *Z. officinale* that act as capping agents [59]. The presence of alkenes in the ginger extract may interfere with the synthesis of ZnONPs [60]. The shift at 1118.51 cm^−1^ (ZnONPs and CuNPs) may be due to the involvement of alcohols, acetate, ethers and –C–O groups of polyols (flavones, terpenoids, and polysaccharides) in the ginger extract. The marked change at 1042 cm^−1^ (AgNPs, ∆ 25 cm^−1^; CuNPs, ∆ 8 cm^−1^) with an increase in the intensity confirmed the role of S = O sulfoxide in the synthesis of NPs. The bands at 865, 1105, 1493, 2929, 3445, and 3836 cm^−1^were attributed to –CH, C–OH, CH_2_–, OCH_3_/CH_2_–CH_3_ and OH functional groups in the alkaloids 6-shogal, 6-gingerol, and α-zingeberene [31]. Moreover, sesquiterpenes such as curcumene, farnesene, geranyl acetate, terpineol, terpenes, geraniols, limonene, and β-bisabolene of *Z. officinale* could be involved in the reduction of metal ions and synthesis of NPs [42]. Shifts at 620.00 cm^−1^ (in all NPs), and 566.00 cm^−1^ (ZnONPs), new bands at 457.05 cm^−1^ (CuNPs) and 464.76 cm^−1^ (AgNPs) suggest the involvement of the metal-O stretching vibration and C–S stretching modes of sulfur-bearing residues [61]. These C–S stretching modes of sulfur-bearing residues confirmed the role of cysteine and methionine in NPs synthesis. The soluble elements in ginger extract could have acted as a stabilizing agent preventing the agglomeration of NPs in solution [62].

From FTIR spectra the total shifts (in descending manner) were ~141(ZnONPs), 96 (AgNPs), and 84 cm^−1^ (CuNPs). Carbonyl groups from amino acid residues, sulfur-bearing residues, phosphorus, P = S, heterocyclic compounds, alcohols, ethers, and acetate played a significant role in the synthesis and stabilization of NPs. The FTIR spectroscopic study revealed that amino acids and peptides form a coating covering the NPs.

### 3.2. Antimicrobial Activity of Synthesized NPs

**Figure 4:**
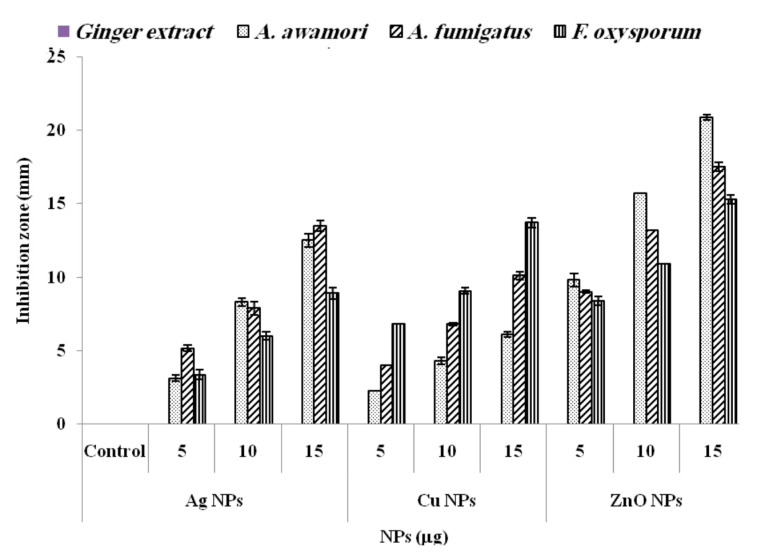
Zone of inhibition (ZOI) of AgNPs, CuNPs and ZnONPs against *Aspergillus awamori* JQ695830.1, *A. fumigatus* JX006238, and *F.oxysporum* FR11 treated with 5, 10, and 15 μg of AgNPs, CuNPs, and ZnONPs synthesized by aqueous extract of *Zingiber officinale*.

From the results, the most effective antifungal agent was ZnONPs. Compared to ginger extract used as negative control and had no antifungal effect, ZnONPs displayed a strong antifungal activity against *A. awamori* with ZOI 20.9 ± 0.35 mm followed by *A. fumigatus* (17.5 ± 0.40 mm) and *F. oxysporum* (15.3 ± 0.20 mm) (Figure 4). Moreover, AgNPs and CuNPs showed the highest antifungal activity against *A. fumigatus* (15.5 ± 0.46 mm) and *F. oxysporum* (13.7 ± 0.33 mm), respectively. ZnONPs inhibited the pigmentation and conidial formation in *A. awamori*. ZnO NPs suppress the germination rate of *Al. alternata*, *Rhizopus stolonifer*, *F. oxysporum*, and *Mucor plumbeus* spores [63]. *A. awamori* was susceptible to ZnONPs and showed the lowest MIC (24.7 ± 1.65 μg/mL) as compared to the other studied species. Moreover, *A. fumigatus* and *F. oxysporum* were sensitive to AgNPs (MIC 30 ± 2.88 μg/mL) and CuNPs (MIC 31.7 ± 1.77 μg/mL) (Table 1). Interestingly, the MIC values of the biosynthesized NPs against the mycotoxigenic fungi are lower than reference antibiotic amphotericin B.

**Figure 5:**
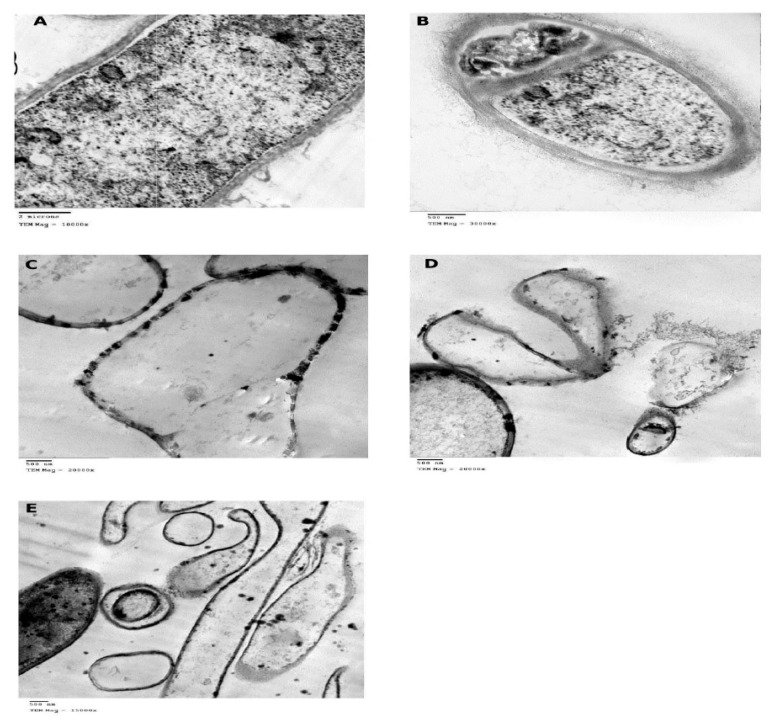
Transmission electron microscope (TEM) micrographs of (**A**,**B**) native *Aspergillus awamori* JQ695830.1 cells and (**C**–**E**) ZnONPs-treated *A. awamori*.

The TEM micrographs of control and ZnONPs-treated *A. awamori* were shown in Figure 5A–E. Ultrathin sections of control cells revealed the cell wall and septum with 700 and 230 nm in thickness, respectively, normal plasma membrane and well-organized cytoplasm with few electron-dense areas (Figure 5A,B). TEM micrographs of ZnONPs-treated *A. awamori* cells revealed the presence of NPs within the cell wall layers, septum, and attached to cell surfaces indicating their affinity towards *A. awamori*. The septum became very thin (immeasurable) (Figure 5C,D). The cell wall became thinner (140 nm) with the complete absence of extracellular fibrillar material, revealing the enhanced permeability of fungal membrane and internalization of NPs. The ruptured cell wall and leakage of the cellular contents was observed (Figure 5D,E). The fungicidal activity of ZnONPs could be explained. Firstly, potential formation of hydrogen bonds between oxygen atoms of ZnONPs and hydroxyl groups of cellulose molecules of fungi, generating H_2_O_2_. Secondly, membrane injury by the abrasive surface of ZnONPs which has defects such as edges and corners [64]. Thirdly, the expression of stress response genes, superoxide dismutase, and glutathione S-transferase were significantly induced by exposure to ZnONPs and ions leading to generation of reactive species (ROS) [65]. Nanoparticles and their ions show a genotoxic effect and destroy DNA. They interact with proteins containing –SH and cause protein denaturation. More than one mechanism can work simultaneously [66].

### 3.3. Effect of ZnONPs on the Amount of OTA Produced by A. awamori in YES Broth

**Figure 6:**
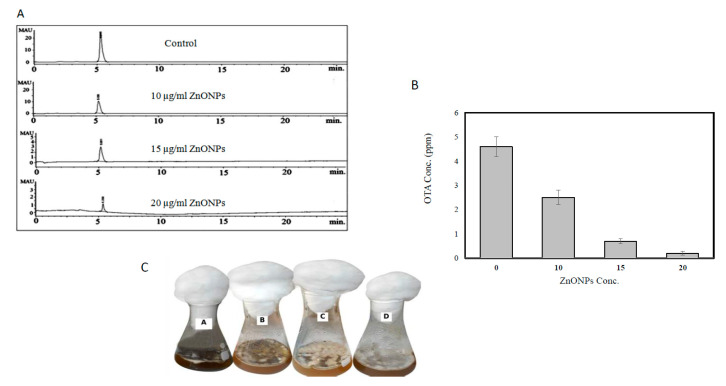
(**A**) HPLC chromatogram of Ochratoxin A from *A. awamori* after treatment with 0, 10, 15, 20 μg of ZnONPs/mL. (**B**) Ochratoxin A yield from *A. awamori* in response to different doses of ZnONPs. (**C**), Visual observation of broth cultures of *A. awamori* JQ695830.1 in response to different doses of ZnONPs.

From HPLC analysis, the ZnONPs inhibited OTA production of *A. awamori* in a concentration-dependent manner (Figure 6A). The concentration of OTA was 4.6 ppm (control), 2.5 ppm (10 μg of ZnONPs/mL), 0.7 ppm (15 μg of ZnONPs /mL) and 0.2 ppm (20 μg of ZnONPs/mL), with reduction percentages of 45.6, 84.7, and 95.6%, respectively (Figure 6B). Obviously, the gradual reduction in OTA has been observed to be correlated with inhibition in the formation of *A. awamori* conidia (Figure 6C). The sporulation was more sensitive to ZnONPs stress than vegetative growth where the inhibition percentage in the dry weight was 12.5% (10 μg of ZnONPs/mL), 53.13% (15 μg of ZnONPs /mL), and 75.01% (20 μg of ZnONPs/mL). The reproduction of *Aspergillus* spp. involves mainly the formation of spores which are resistant to unfavorable conditions and help in the spread of this toxigenic fungal pathogen [67,68]. The spore production in *Trichoderma harzianum* was more sensitive to AgNPs than mycelial growth. *A. awamori* regarded as a fungal pathogen of garlic (*Allium sativum* L.) and caused black gill infection on Pacific white shrimp (*Litopenaeus vannamei*) [69]. The ZnONPs reduced mycotoxins production in *F. oxysporum*, *P. expansum*, *A. ochraceus* and *A. niger* [70,71]. On the other hand, it was stated that citrinin biosynthesis was induced in *P. verrucosum* when it was subjected to oxidative stress [53]. A relation between sporulation and mycotoxin production has been recorded in several mycotoxigenic genera. Mycotoxins secreted by fungal colonies at the approximate time of sporulation [52].

In conclusion, the biosynthesis of AgNPs, CuNPs, and ZnONPs using *Z. officinale* aqueous extract provides a fast, low-cost, single-step, effective and ecofriendly approach. Visual observations, UV–Vis, TEM, FTIR, ζ-potential, and XRD techniques confirmed the formation of NPs by *Z. officinale* aqueous extract. The FTIR analyses and protein contents confirmed the significant role of proteins in the capping and stabilization of NPs, especially in the case of ZnONPs. The NPs showed high antifungal activity by the disk diffusion method. ZnONPs inhibit the sporulation and OTA production by *A. awamori*. The phytosynthesized ZnONPs have more useful physical properties than the chemically synthesized NPs, that could open a new avenue for further applications of biosynthesized NPs.

## Figures and Tables

**Table 1:** Minimum inhibition concentration (MIC) ± SD of AgNPs, CuNPs, and ZnONPs, synthesized by aqueous extract of *Zingiber officinale* Roscoe and amphotericin B.

Fungal Isolates	MIC (μg/mL)			
	AgNPs	CuNPs	ZnONPs	Amphotericin B
*A. awamori*	33.3 ± 1.67	31.7 ± 1.77	24.7 ± 1.65	25.33 ± 2.02
*A. fumigates*	30 ± 2.88	38.3 ± 1.50	26.7 ± 1.70	26.52 ± 1.34
*F. oxysporum*	35 ± 2.80	31.7 ± 1.36	28.3 ± 1.40	22.11 ± 1.81

Standard deviation ± SD.

## Data Availability

All the obtained data are included on this paper.
